# Hepatic Methionine Homeostasis Is Conserved in C57BL/6N Mice on High-Fat Diet Despite Major Changes in Hepatic One-Carbon Metabolism

**DOI:** 10.1371/journal.pone.0057387

**Published:** 2013-03-05

**Authors:** Christoph Dahlhoff, Charles Desmarchelier, Manuela Sailer, Rainer W. Fürst, Alexander Haag, Susanne E. Ulbrich, Björn Hummel, Rima Obeid, Jürgen Geisel, Bernhard L. Bader, Hannelore Daniel

**Affiliations:** 1 Biochemistry Unit, PhD Group, Research Center for Nutrition and Food Sciences, Technische Universität München, Freising-Weihenstephan, Germany; 2 Epigenetics, Imprinting and Nutrition, Research Center for Nutrition and Food Sciences, Technische Universität München, Freising-Weihenstephan, Germany; 3 Nutritional Medicine Unit, Research Center for Nutrition and Food Sciences, Technische Universität München, Freising-Weihenstephan, Germany; 4 Physiology Unit, ZIEL, Research Center for Nutrition and Food Sciences, Technische Universität München, Freising-Weihenstephan, Germany; 5 Clinical Chemistry and Laboratory Medicine/Central Laboratory^,^ University Hospital of the Saarland, Homburg, Germany; University of California San Francisco, United States of America

## Abstract

Obesity is an underlying risk factor in the development of cardiovascular disease, dyslipidemia and non-alcoholic fatty liver disease (NAFLD). Increased hepatic lipid accumulation is a hallmark in the progression of NAFLD and impairments in liver phosphatidylcholine (PC) metabolism may be central to the pathogenesis. Hepatic PC biosynthesis, which is linked to the one-carbon (C1) metabolism by phosphatidylethanolamine N-methyltransferase, is known to be important for hepatic lipid export by VLDL particles. Here, we assessed the influence of a high-fat (HF) diet and NAFLD status in mice on hepatic methyl-group expenditure and C1-metabolism by analyzing changes in gene expression, protein levels, metabolite concentrations, and nuclear epigenetic processes. In livers from HF diet induced obese mice a significant downregulation of cystathionine β-synthase (CBS) and an increased betaine-homocysteine methyltransferase (BHMT) expression were observed. Experiments *in vitro*, using hepatoma cells stimulated with peroxisome proliferator activated receptor alpha (PPARα) agonist WY14,643, revealed a significantly reduced Cbs mRNA expression. Moreover, metabolite measurements identified decreased hepatic cystathionine and L-α-amino-n-butyrate concentrations as part of the transsulfuration pathway and reduced hepatic betaine concentrations, but no metabolite changes in the methionine cycle in HF diet fed mice compared to controls. Furthermore, we detected diminished hepatic gene expression of *de novo* DNA methyltransferase 3b but no effects on hepatic global genomic DNA methylation or hepatic DNA methylation in the Cbs promoter region upon HF diet. Our data suggest that HF diet induces a PPARα-mediated downregulation of key enzymes in the hepatic transsulfuration pathway and upregulates BHMT expression in mice to accommodate to enhanced dietary fat processing while preserving the essential amino acid methionine.

## Introduction

The epidemic occurrence of obesity in the general population has caused an increase in the incidence of obesity-associated diseases. NAFLD is an incremental liver dysfunction that is associated with obesity [1] and induces a wide range of hepatic alterations starting with steatosis and non-alcoholic steatohepatitis that can progress to cirrhosis and hepatocellular carcinoma [2]. In general, diet-induced obesity (DIO) in mice generated by feeding animals a HF diet causes hyperglycemia, hyperinsulinemia, reduced glucose tolerance and hepatic triacylglycerol (TG) accumulation [3], [4]. Previously, we have shown that feeding C57BL/6N mice a beef tallow based HF diet resulted in significant changes in hepatic and intestinal phospholipid (PL) and cholesterol contents, as well as changes in PC signature indicative for a) an increased PC synthesis via the CDP-choline pathway, b) an increased phosphatidylethanolamine (PE) methylation pathway activity in the liver and c) alterations in membrane PL remodeling [5]. The observed higher levels of PC species with longer carbon chains found in the liver could originate most likely from an increased activity of the PE methylation pathway in hepatocytes [6]. Changes of specific PC levels upon HF diet may modulate the activation state of the nuclear receptor PPARα, which is a prime candidate promoting fatty acid oxidation, lipid transport and ketogenesis in liver and intestine. Diacyl-phosphatidylcholine PC.aa (16∶0/18∶1) was recently identified as a natural ligand and activator of PPARα [7].

Biosynthesis and turnover of PC are important in the formation of VLDL particles and lipid export from hepatocytes which, when disturbed, promotes the accumulation of lipid droplets in hepatocytes causing steatosis [8], [9], [10]. Hepatic PC biosynthesis is mainly dependent on dietary choline supply via the CDP-choline pathway, which accounts for approximately 70% of hepatic PC biosynthesis, whereas the remaining 30% is synthesized by the methylation of PE via phosphatidylethanolamine N-methyltransferase (PEMT) [9]. This second pathway is also known to be required for VLDL secretion [11], [12]. Interestingly, PEMT-deficient mice (Pemt^−/−^) fed a HF diet are protected from DIO due to disturbed *de novo* choline biosynthesis (PE methylation pathway), thus linking PC biosynthesis to the development of DIO [13]. Furthermore, dietary choline supplementation of Pemt^−/−^ mice reversed the protective effect suggesting that choline is essential for systemic lipid metabolism and distribution [13]. Pemt polymorphisms resulting in altered PEMT activities have also been associated with the susceptibility for NAFLD in humans [14]. Moreover, an enhanced secretion of PC derived from the hepatic PE methylation pathway has been observed in mice fed a high-fat/high-cholesterol diet [15]. This may indicate increased demands of PL secreted into bile for the assembly of micelles [16] required in intestinal fat absorption.

**Figure 1:**
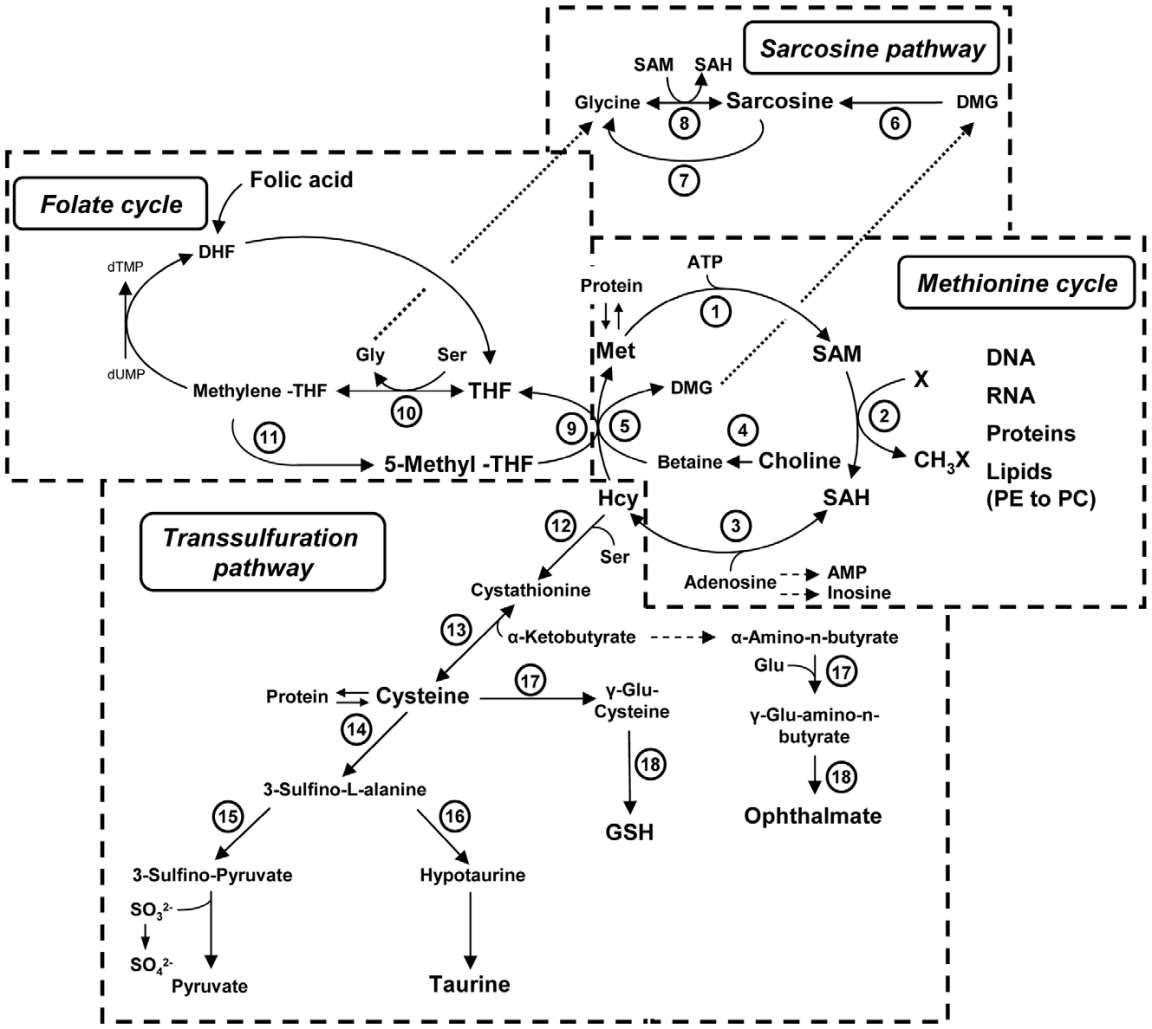
Hepatic C1-metabolism with pathways and operating enzymes. Enzymes of the methionine cycle, folate cycle, sarcosine pathway and transsulfuration pathway are numbered and depicted as follows: 1, MAT, Methionine adenosyltransferase; 2, SAM-dependent methyltransferase; 3, AHCY, S-adenosylhomocysteine hydrolase; 4, CHDH, Choline oxidase and BADH, Betaine aldehyde dehydrogenase; 5, BHMT, Betaine-homocysteine methyltransferase; 6, DMGDH, Dimethylglycine dehydrogenase; 7, SARDH, Sarcosine dehydrogenase or PIPOX, Pipecolic acid oxidase; 8, GNMT, Glycine methyltransferase; 9, MTR, Methionine synthase; 10, SHMT1/2, Serine hydroxymethyltransferase 1 or 2; 11, MTHFR, 5,10-methylenetetrahydrofolate reductase; 12, CBS, Cystathionine β-synthase; 13, CTH, Cystathionase; 14, CDO1, Cysteine dioxygenase 1; 15, GOT1, Glutamate oxaloacetate transaminase 1; 16, CSAD, Cysteine sulfinic acid decarboxylase; 17, GCL, Glutamate-cysteine ligase; 18, GSS, Glutathione synthetase.

The C1-metabolism is the principal pathway providing the methyl-donor S-adenosyl-methionine (SAM) in the methionine cycle necessary for numerous transmethylation reactions (**Fig. 1**). PEMT transfers three methyl-groups from SAM to PE thus linking PC biosynthesis and C1-metabolism in the liver [8], [17]. SAM-dependent transmethylation leads to the synthesis of S-adenosyl-homocysteine (SAH) that is hydrolyzed to homocysteine (Hcy). Hcy is either remethylated to methionine via folate-dependent processes in the folate cycle and by choline oxidation processes associated with the sarcosine pathway or converted via the transsulfuration pathway to cystathionine which can be catabolized to cysteine [8]. Cysteine can be used for glutathione synthesis or is further metabolized (**Fig. 1**) to either taurine as main metabolic end product or used up for sulfate production [8]. Genetic studies in Pemt^−/−^ mice and CTP:phosphocholine cytidyltransferase 1α gene knockout mice (CTα^−/−^) deficient for the hepatic PEMT pathway or CDP-choline pathway, respectively, demonstrate a functional link between C1-metabolism, transmethylation processes and PC biosynthesis. Pemt^−/−^ mice display only around 50% of Hcy plasma levels compared to the levels found in wild type mice [18], whereas CTα^−/−^ mice show elevated (20 to 40%) plasma Hcy levels [19].

Considering the relations described between (i) the C1-metabolism and PC synthesis, (ii) observed changes in PL contents and PC signatures upon HF diet feeding and (iii) the activation of PPARα by a diacyl-phosphatidylcholine identified as a natural PPARα ligand, the following questions arise: how does a high dietary fat load impact on the hepatic C1-metabolism pathways at the levels of gene and protein expression as well as metabolites? Which role plays PPARα signaling? Can alterations in important transmethylation processes, such as DNA methylation, be observed?

To answer these questions, we analyzed gene and protein expression levels and metabolite concentrations in the liver of animals on HF and control diets. Furthermore, we examined whether PPARα is involved in the observed changes in obese mice by *in vitro* experiments. Since methyl-group homeostasis may also affect epigenetic processes, we measured hepatic gene expression level of DNA methyltransferases (Dnmts) and determined global and local genomic DNA methylation in livers from mice of both dietary groups.

## Materials and Methods

### Animal Study and Sample Collection

Eight-week-old male C57BL/6NCrl mice (Charles River Laboratories) were maintained in 12 h light/dark cycles with unlimited access to food and water. The dietary intervention was performed as previously described [5]. Briefly, after 2 weeks feeding a standard laboratory chow (Ssniff GmbH, no. V1534), mice with similar mean body weight were divided randomly into a control (C) and a HF group (n = 12). The C group was given a carbohydrate−/starch-based diet, comprising 4.2% (w/w) fat (Ssniff GmbH, no. E15000-04; **Table S1**), while the HF group was given a beef tallow-based diet, with 34% (w/w) fat (Ssniff GmbH, no. E15741-34; **Table S1**) for 12 weeks. Formulations of the individual diets were made from components with the same lot number. Food intake was measured during the feeding trial and the corresponding intake of energy, protein, methionine, cystine, choline chloride, folate and vitamin B_12_ were calculated for twelve weeks of feeding (**Table S2**).

At the end of the feeding trial, mice in a non-fasting state were anesthetized using isoflurane and blood was taken from the retro-orbital sinus. After killing the mice by cervical dislocation, liver samples were collected, snap-frozen in liquid nitrogen and stored at −80°C until analysis. Animal experiments were done in accordance with the German guidelines for animal care and approved by the Department of Veterinary Affairs of the government of Oberbayern/Germany.

#### Cell culture

Fao rat hepatoma cells were originally cloned and characterized by Deschatrette and Weiss [20] and available at the European Collection of Cell Cultures (EACC, Salisbury, Wiltshire,UK; ECACC catalog code: 89042701). Fao rat hepatoma cells (obtained from T. Skurk, Technische Universität München, Nutritional Medicine, Freising, Germany) were cultured in Dulbecco’s Modified Eagle Medium (DMEM 25 mM glucose, Invitrogen) supplemented with 10% fetal calf serum (PAA), 100 U/ml penicillin and 100 µg/ml streptomycin (PAA) in a humidified atmosphere of 95% air and 5% CO_2_ at 37°C. Two days before the experimental start and prior reaching confluence, cells were preincubated with DMEM (5.56 mM glucose, Invitrogen) supplemented with 0.5% bovine serum albumin (Sigma-Aldrich), followed by stimulation with different concentrations of the PPARα agonist WY14,643 (Sigma-Aldrich) dissolved in DMSO/PBS (50∶50) as vehicle for 24 h. Fao rat hepatoma cells, treated only with vehicle (without PPARα agonist) were used as non-stimulated control cells. Cells were harvested for RNA isolation using Trizol reagent (Invitrogen).

### RNA Isolation and Real-time Quantitative PCR (RT-qPCR

Isolation of total liver RNA and quantification of mRNA expression by RT-qPCR were performed as previously described [5]. For analysis of Fao rat hepatoma gene expression, 50 ng total RNA was used, and for normalization, the invariant gene β-glucuronidase was chosen. Oligonucleotide primer sequences are listed in **Tables S3 and S4**.

### Analysis of Amino Acids and their Derivatives

Liver tissues were ground in liquid nitrogen and 100 mg of the homogenates were dissolved in 150 µl H_2_O/MeOH (50∶50), vortexed and centrifuged. 40 µl of the supernatant was used to determine the concentration of amino acids and their derivatives following the iTRAQ-labeling method using the AA45/32™ Kit (Applied Biosystems). Samples were treated according to the manufacturer‘s instructions and analyzed via LC-MS/MS (3200QTRAP LC/MS/MS, Applied Biosystems). Spectra were processed using the Analyst® 1.5 Software [21]. Protein determination for data normalization was performed using the Bradford assay (Biorad) with BSA as standard.

Hepatic betaine, dimethylglycine (DMG) and choline concentrations were analyzed by LC-MS/MS according to Holm *et al*. [22] with minor modifications. 5 µl liver homogenates prepared and dissolved in H_2_O/MeOH (50∶50) as described above were used to determine the concentration of hepatic betaine, DMG and choline by LC-MS/MS (QTRAP5500, ABSCIEX). As internal standard d_11_-betaine (Sigma-Aldrich) was used.

The HPLC-MS/MS detection of hepatic SAM and SAH levels was performed using a Waters 2795 alliance HT, coupled to a Quattro Micro API tandem mass spectrometer (Waters Corporation) according to the previously described methodology with minor modifications [23]. After homogenization of liver tissues in 1 N acetic acid and centrifugation at 12000 g for 10 min, 25 µl of the supernatants were added to 375 µl of 20 mM ammonium acetate buffer (pH 7.4) and 40 µl of internal standard (SAM and SAH), cleaned on SPE column and analyzed by HPLC-MS/MS.

Liver Hcy and cystathionine were analyzed by gas chromatography mass spectrometry (GC-MS) according to Stabler *et al.*, with minor modifications [24]. Briefly, liver tissue samples were homogenized with 0.1 M perchloric acid, centrifuged at 12000 g for 10 min, neutralized with 1 M K_3_PO_4_, and stored for final preparation as described by Stabler *et al.* [24].

#### Protein extraction and western immunoblot analysis

Western immunoblot analysis was performed as previously described with minor modifications [25]. Liver tissues were homogenized in RIPA supplemented with 1% protease inhibitor cocktail and 1% phosphatase inhibitor cocktail II (Sigma). Hepatic liver protein content calculated as hepatic protein per 1 g liver tissue was similar in control (127.5±10.1) and HF mice (118.3±6.1). Total protein extracts were separated by SDS-polyacrylamide gel electrophoresis and transferred to a nitrocellulose membrane (Whatman GmbH). For immunodetection, membranes were incubated with the following primary antibodies at 1∶1000 to 1∶20000 dilutions in TBS-T at 4°C over night: goat anti-BHMT (Novus Biologicals), rabbit anti-CBS (Aviva Systems Biology), rabbit anti-β-actin and rabbit anti-histone H3 (Cell Signaling). After several washing steps, secondary donkey anti-goat or goat anti-rabbit conjugated with IRDye 800 (Li-cor) antibodies at 1∶10000 to 1∶50000 dilutions in TBS-T were exposed at room temperature for 1 h. Signal detection and quantification of fluorescence intensity were performed with the Odyssey infrared imaging system (Li-cor) and by using the Odyssey Application Software 3.0 (Li-cor) for calculation of integrated intensity. Histone H3 and β-Actin were used for normalization of protein abundance.

### DNA Isolation

Genomic DNA was extracted from liver of C and HF diet fed mice by 12 mAU proteinase K digestion over night and 200 µg RNaseA incubation for 2 min followed by chloroform extraction. DNA was isolated from the aqueous phase using the DNeasy Blood & Tissue Kit (Qiagen) according to the manufacturer’s protocol. DNA concentration was determined on a NanoDrop ND-1000 UV-Vis spectrophotometer (NanoDrop Technologies).

### Analysis of Global Genomic DNA Methylation Using Luminometric Methylation Assay (LUMA)

Global DNA methylation was quantified using LUMA as previously described [26], [27] with minor variations. Briefly, 1 µg genomic DNA was cleaved with the FastDigest® restriction enzymes *Hpa*II+*Eco*RI or *Msp*I+*Eco*RI (Fermentas) in two separate reactions for 15 min. The digestions were performed in a 96-well format using a PSQ96™ MA system (Biotage AB). Peak luminometric heights were calculated with the PSQ96™ MA software. The *Hpa*II/*Eco*RI and *Msp*I/*Eco*RI ratios were calculated as (dGTP+dCTP)/dTTP for the respective reactions. DNA methylation was calculated from the (*Hpa*II/*Msp*I) ratio at the investigated sites.

### Methylation-sensitive Quantitative PCR (MS-qPCR)

Local genomic DNA methylation of CpG sites was quantified using MS-qPCR as previously described [28], [29] with minor modifications. For each digestion, 1 µg DNA was incubated either without enzyme (sham) or with the methylation-sensitive FastDigest® restriction enzymes (Fermentas) *Aci*I or *Hpa*II at 37°C for 5 h, followed by heat inactivation at 65°C for 20 min. For DNA methylation analysis, 5 ng of digested genomic DNA were amplified using the QuantiTect® SYBR Green PCR Kit (Qiagen) on a Mastercycler ep realplex apparatus (Eppendorf) with CpG island specific primer pairs flanking informative restriction sites (*Aci*I, *Hpa*II). Following conditions were used: 1 initial cycle at 95°C for 15 min followed by 40 cycles of denaturation, annealing and elongation (at 95°C for 15 s, 61°C for 30 s and 72°C for 30 s, respectively), subsequently the melting curve analysis (1.75°C/min) was performed. For normalization, an internal control region on chromosome 18 was amplified using a primer pair flanking no informative restriction site [28], [29]. MS-qPCR primer sequences are shown in **Table S5**. C_q_-values were retrieved from the realplex 2.0 software (Eppendorf). Calculation of restriction site DNA methylation was performed with the modified equation 100*2 ^−(ΔCq ROI − ΔCq control)^ [28], [29].

### Bisulfite Genomic Pyrosequencing

Bisulfite conversion of genomic DNA was performed as previously described [30]. Briefly, 500 ng genomic DNA and the EZ DNA Methylation-Gold Kit (Zymo Research) was used according to the manufacturer’s instructions. For quantification of individual CpG-site methylation, 35 ng of bcDNA was amplified on a Rotor-gene Q (Qiagen) using the EpiTect HRM PCR Kit (Qiagen) with specific primer pairs (**Table S6**) and the following thermal cycling conditions: at 95°C for 5 min, followed by 50 cycles at 95°C for 10 s, at 58°C for 30 s and at 72°C for 30 s, respectively. Specificity was controlled by high resolution melting curve analysis with 0.05°C/2 s increments. The PCR products were extracted using the Wizard SV Gel and PCR clean-Up System (Promega) according to the manufacturer’s instructions. For subsequent pyrosequencing the biotinylated strand was isolated and released into annealing buffer containing the specific sequencing primer (**Table S6**). Determination of local genomic DNA methylation was performed on a PyroMark Q24 System (Qiagen) and all pyrosequencing reagents (Qiagen) were prepared according to the manufacturer’s instructions. Calculation of individual CpG-site methylation was performed by the PyroMark Q24 Software (Qiagen).

### Statistical Analysis

Results are presented as mean ± SEM. Statistical analyses were performed using Prism 4.01 software (GraphPad Software). Data were tested using Student’s t-test. In case of inhomogeneous variances, data were analyzed using Student’s t-test with Welch’s correction. The *in vitro* experiment was analyzed using one-way ANOVA with tukey’s test used as post hoc test. For all tests, the bilateral alpha risk was α = 0.05. Differences in liver amino acid concentrations determined with the iTRAQ-labeling method were tested using the R version 2.9.2 (R Foundation of Statistical Computing) [31]. The p-values were adjusted for multiple testing using the *p.adjust* function within the R-library *limma* and the Benjamini-Hochberg method [32].

## Results

### Alterations in Gene Expression of Enzymes Operating in the Hepatic Transsulfuration and Sarcosine Pathways

**Figure 2:**
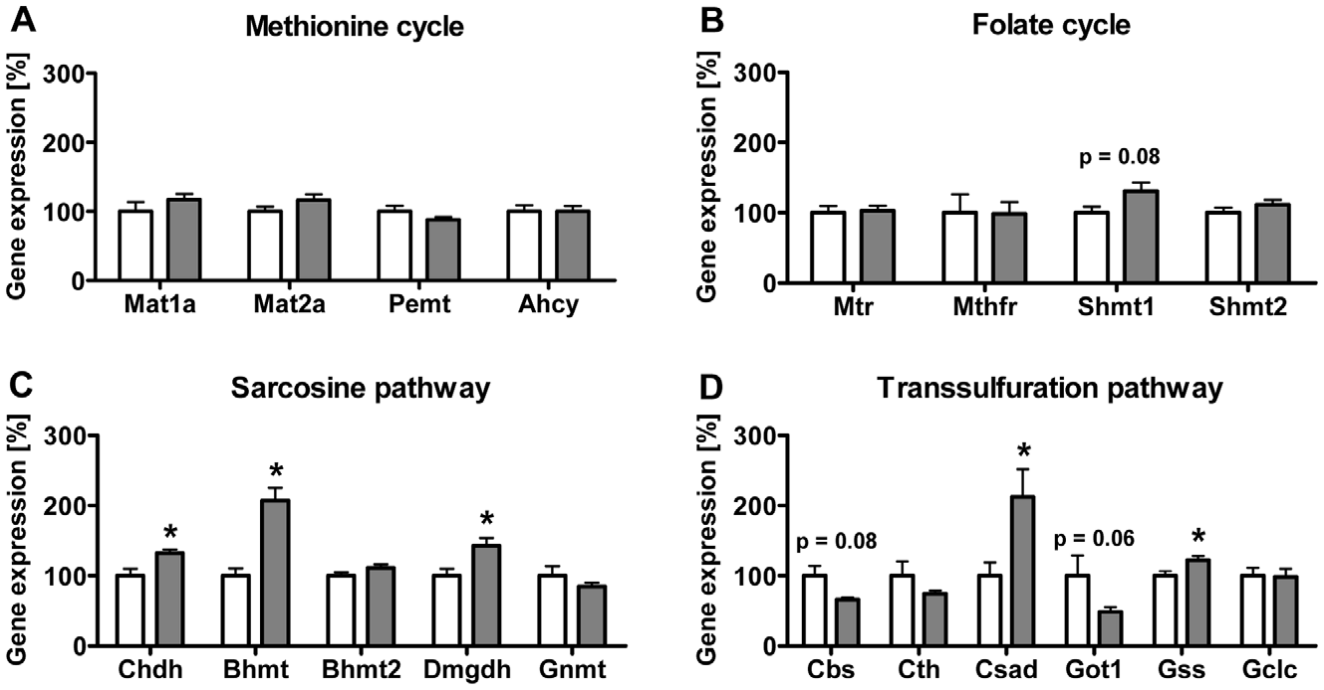
Effect of HF feeding on hepatic mRNA expression levels of C1-metabolism enzymes. Data are presented as mean ± SEM (n = 5–6). Analysis of the methionine cycle (**A**), the folate cycle (**B**), sarcosine pathway (**C**), and transsulfuration pathway (**D**). Open and grey bars represent data from animals fed control and HF diet, respectively. Asterisk indicates statistical significance (p<0.05).

In a previous study, we have analyzed the development of obesity, insulin resistance and hepatic steatosis, induced by HF diet feeding, in C57BL/6N mice and detected significant alterations of hepatic PL homeostasis and specific PC signature [5]. Here, we asked whether obesity and NAFLD status influence hepatic methl-group expenditure and C1-metabolism. To detect the impact of HF diet feeding and NAFLD status on hepatic mRNA expression of key enzymes in the C1-metabolism, we performed RT-qPCR analysis using total RNA of liver tissues from these obese and control mice. The first set of selected genes analyzed (**Fig. 1**) comprised genes involved (i) directly in the synthesis of SAM such as methionine adenosyltransferase I alpha (Mat1a) and methionine adenosyltransferase II alpha (Mat2a), (ii) in SAH hydrolysis or transmethylation processes such as the S-adenosylhomocysteine hydrolase (Ahcy) and Pemt and (iii) in folate-dependent homocysteine remethylation like the 5-methyltetrahydrofolate-homocysteine methyltransferase (Mtr) and methylenetetrahydrofolate reductase (Mthfr). Surprisingly, no significant changes in expression levels were detected for this group of genes (**Fig. 2A, B**). Serine hydroxymethyltransferase 1 (Shmt1) mRNA levels were increased by 30.7±12.0% (p = 0.08) in obese mice over controls but serine hydroxymethyltransferase 2 (Shmt2) remained unaffected by dietary treatment (**Fig. 2B**). However, in the second set of genes representing the choline oxidation and sarcosine pathway, HF diet feeding resulted in higher mRNA expression levels of genes involved in the betaine-dependent remethylation of Hcy. This included Bhmt with expression levels elevated by 107.3±17.4%; (p = 0.001), while betaine-homocysteine methyltransferase 2 (Bhmt2) was not significantly affected (p = 0.16). The choline dehydrogenase (Chdh) increased by 32.0±5.3% (p = 0.017), and the dimethylglycine dehydrogenase precursor (Dmgdh) mRNA level was elevated by 43.3±10.6% (p = 0.017) compared to control mice (Fig. 2C). The gene expression of glycine N-methyltransferase (Gnmt), important in hepatic SAM-homeostasis, was unaffected (p = 0.29) by the dietary treatment (**Fig. 2C**). For genes operating in the transsulfuration pathway and involved in the taurine and glutathione synthesis (**Fig. 2D**), reduced mRNA levels of the cystathionine β-synthase (Cbs) gene were detected with 66.3±2.7% of controls and marginal significance (p = 0.08), but no significant differences for cystathionase (Cth) were measured (p = 0.29). Whereas mRNA expression levels were significantly elevated for cysteine sulfinic acid decarboxylase (Csad) by 112.5±38.9%, (p = 0.038) and glutathione synthetase (Gss) by 22.4±5.9% (p = 0.032), glutamate-cysteine ligase (catalytic subunit, Gclc) did not reveal regulation (p = 0.92). Glutamate oxaloacetate transaminase 1 (Got1), implicated in the processing of cysteine to sulfate, showed marginally decreased mRNA levels (48.7±7.0% of control; p = 0.06) in HF diet fed mice **(** **Fig. 2D**).

### Hepatic BHMT Protein Levels Increased and CBS Levels Decreased upon HF Diet Feeding

**Figure 3:**
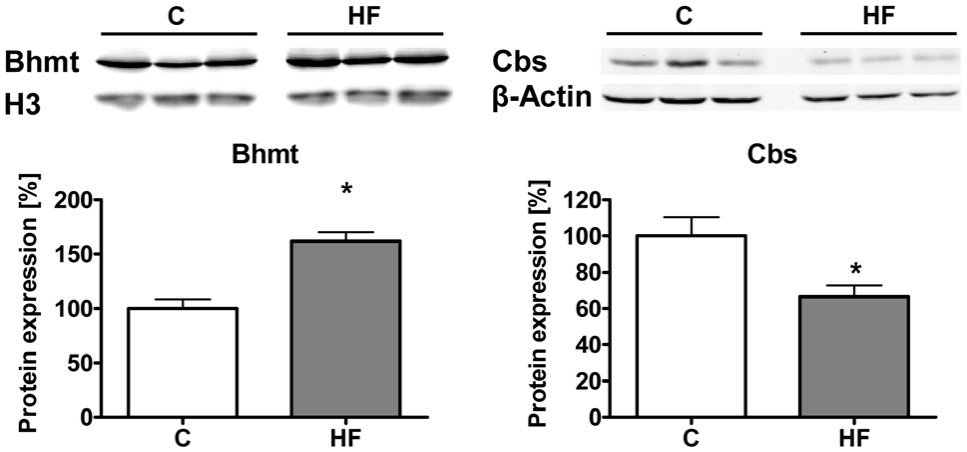
Western blot analysis of hepatic BHMT and CBS protein expression after 12 weeks of feeding. Data are presented as mean ± SEM (n = 6). Open and grey bars represent control and HF animals, respectively. Asterisk indicates statistical significance (p<0.05).

The observed changes in gene expression of the branch-point enzymes BHMT and CBS were further analyzed on protein level by Western blot analysis using liver protein extracts from animals on HF and control diets. As shown in **Figure 3**, BHMT levels were increased by 61.9±8.3% (p<0.001) whereas CBS protein density was decreased in DIO animals to 66.6±6.2% (p = 0.02) of that in controls.

### Metabolite Profiling Data Suggest a Repression of the Hepatic Transsulfuration Pathway

**Figure 4:**
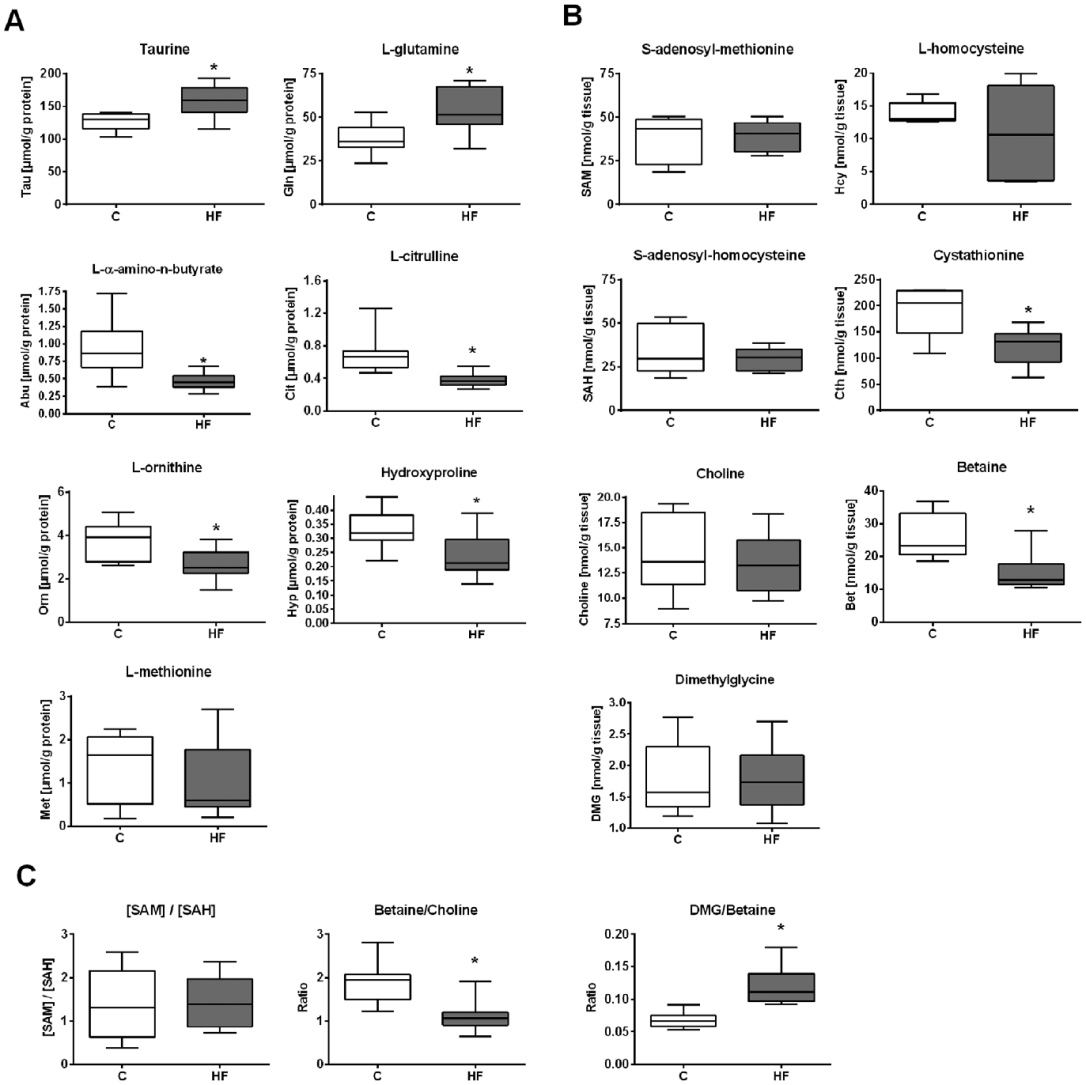
Impact of HF diet on selected hepatic metabolite concentrations after 12 weeks of feeding. Data are presented as box and whisker plot. (**A**) Selected data for taurine, L-glutamine, L-α-amino-n-butyrate, L-citrulline, L-ornithine, hydroxyproline and L-methionine (n = 9–11). (**B**) Analysis of S-adenosyl-methionine, S-adenosyl-homocysteine, L-homocysteine, cystathionine (n = 5–6) and choline, betaine and dimethylglycine (n = 7–9). (**C**) Selected ratios between measured hepatic metabolite concentrations. Open and grey bars represent control and HF mice, respectively. Asterisk indicates statistical significance (p<0.05).

A total of 35 amino acids and their derivatives were quantified by LC-MS/MS, GC-MS/MS in hepatic tissues (**Fig. 4** **, Table S7**). Taurine and glutamine concentrations were elevated in DIO mice by 24.7±5.3% (p = 0.015) and 39.9±9.8%; (p = 0.034), respectively, as shown in **Figure 4A**. Concentrations for L-α-amino-n-butyrate, L-citrulline, L-ornithine and hydroxyproline significantly decreased by 30% or even 50% upon HF diet feeding compared with controls. However, methionine levels remained unaffected (**Fig. 4A**) in obese mice. Concentrations of the methionine cycle metabolites SAM, SAH and Hcy determined by LC-MS/MS and GC-MS, respectively, did not reveal significant differences (**Fig. 4B**), nor did the methylation index [SAM]/[SAH] ratio change in mice fed a HF or control diet (**Fig. 4C**). However, the analysis of cystathionine, a major transsulfuration metabolite, by GC-MS revealed a significantly reduced concentration in obese mice of 64.0±7.7% (p = 0.026) compared with controls, and in the sarcosine pathway the metabolite betaine showed significantly diminished concentration in obese mice of 57.5±5.9% (p<0.001) compared to the controls (**Fig. 4B**). Furthermore, the hepatic [betaine]/[choline] ratio was significantly decreased (p<0.001) and the [dimethylglycine]/[betaine] ratio significantly increased (p<0.001) in HF mice (**Fig. 4C**).

### Increased PPARα Activity may Lead to the Downregulation of the Hepatic Transsulfuration Pathway

**Figure 5:**
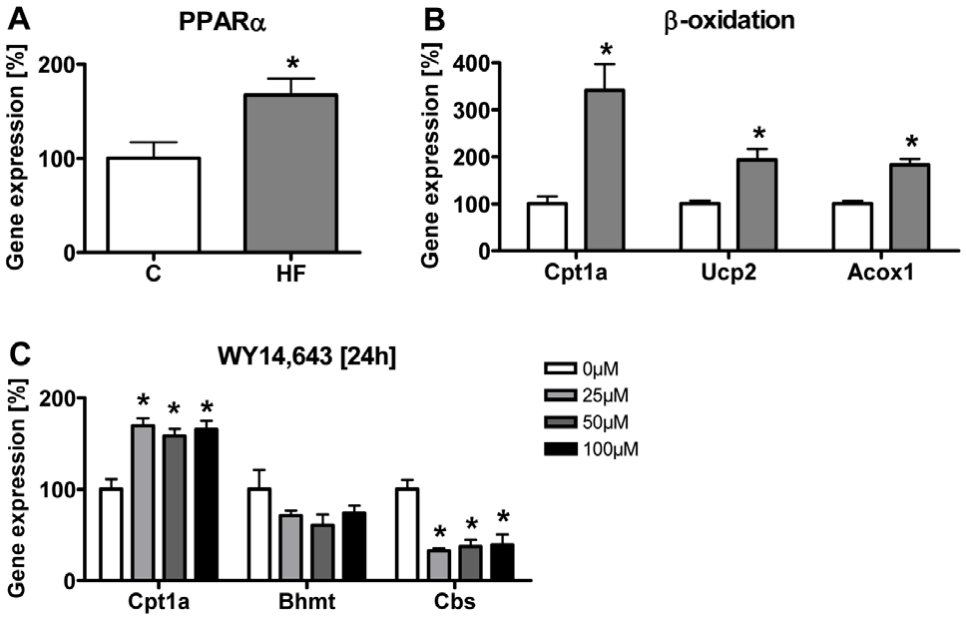
PPARα activity and C1-metabolism associated regulations in obese mice and in rat hepatoma cells (Fao). (**A**) PPARα mRNA expression analysis. (**B**) Analysis of selected PPARα target genes. (**C**) Gene expression analysis of Fao cells stimulated with 25 µM, 50 µM and 100 µM WY14,643 for 24 h. Data are presented as mean ± SEM (n = 5–6 for **A** and **B**; n = 4 for **C**). Open and grey bars represent control and HF animals, respectively (**A**, **B**). Asterisk indicates statistical significance (p<0.05).

Hepatic PPARα is known to critically control gene expression of a large variety of genes involved in lipid metabolism and in particular in ω- and β-oxidation of fatty acids. In addition, PPARα has been suggested to regulate the transsulfuration pathway [33]. To assess whether hepatic PPARα mRNA expression levels were regulated in HF mice, we performed gene expression analysis of PPARα and some of its downstream target genes by RT-qPCR analysis (**Fig. 5A, B**). In comparison with control, PPARα mRNA expression was elevated to 167.4±17.2% (p = 0.022) and selected PPARα target genes also showed significantly higher mRNA levels, including carnitine palmitoyltransferase 1a (Cpt1a) increased to 341.3±16.1% (p = 0.009), uncoupling protein 2 (Ucp2) to 193.0±23.1% (p = 0.012) and acyl-coenzyme A oxidase 1 (Acox1) levels to 182.6±12.6% (p<0.001) suggesting in total an increase in hepatic PPARα activity. To further examine the underlying mechanisms causing an upregulation of BHMT and a downregulation of CBS, we explored whether PPARα-activation by the agonist WY14,643 in rat hepatoma cells (Fao) can cause similar changes in gene expression *in vitro* as observed in the livers of obese mice. Treatment of Fao cells with increasing concentrations of WY14,643 for 24 h indeed augmented the mRNA levels of the PPARα target gene Cpt1a (25 µM, 169.4±8.3%; 50 µM, 158.4±7.7%; 100 µM, 165.3±9.4%; p<0.001) and reduced the mRNA of Cbs (25 µM, 32.7±2.6%; 50 µM, 37.4±7.5%; 100 µM, 39.1±11.7%; p<0.001) but did not change Bhmt mRNA expression (p = 0.24) in comparison with untreated control cells (**Fig. 5C**).

### Hepatic DNA Methyltransferase 3b Gene Expression, but not Hepatic Global Genomic DNA Methylation Levels, is Altered in DIO Mice

**Figure 6:**
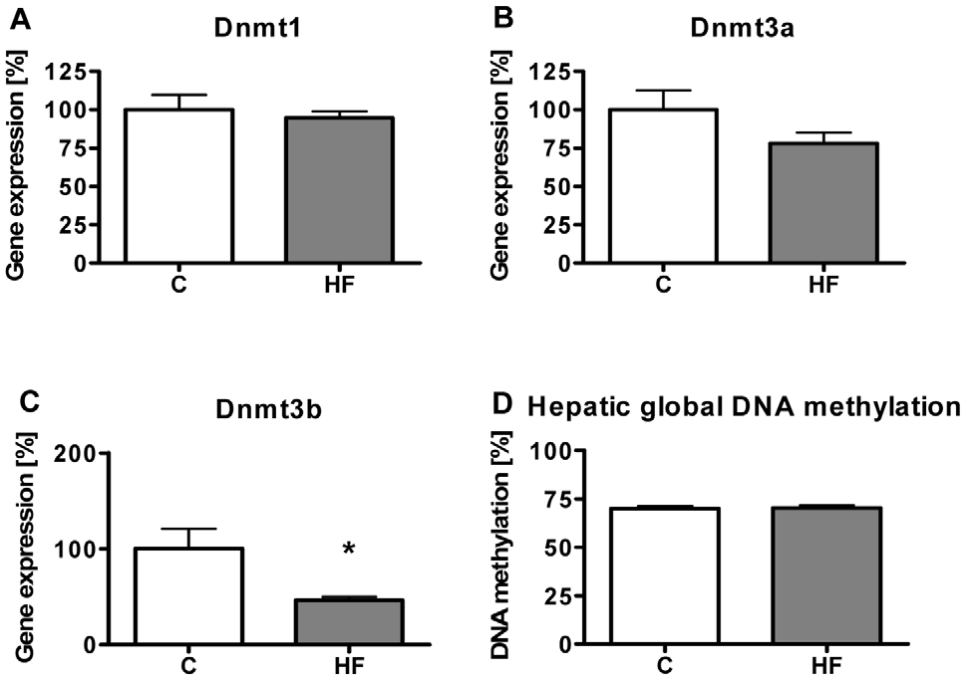
Influence of HF diet on hepatic Dnmt gene expression and global DNA methylation. (**A–C**) Quantification of Dnmt gene expression after 12 weeks of feeding (n = 5–6). Control mice showed stronger expression for Dnmt1 (Ct = 25.13±0.06) and *de novo* Dnmt3a (Ct = 28.10±0.15) than for *de novo* Dnmt3b (Ct = 30.55±0.31). Upon HF feeding, Dnmt1 mRNA expression was unaltered (Ct = 25.03±0.11), but gene expression of Dnmt3a (Ct = 28.28±0.18) and Dnmt3b (Ct = 31.37±0.14) decreased, respectively. (**D**) Analysis of hepatic global DNA methylation of control and HF animals (n = 6). DNA methylation was calculated from the (*Hpa*II/*Msp*I) ratio, whereby a ratio of 1 indicates 0% methylation and a ratio approaching 0 corresponds to 100% DNA methylation at the investigated sites. Data are presented as mean ± SEM. Open and grey bars represent control and HF animals, respectively. Asterisk indicates statistical significance (p<0.05).

**Figure 7:**
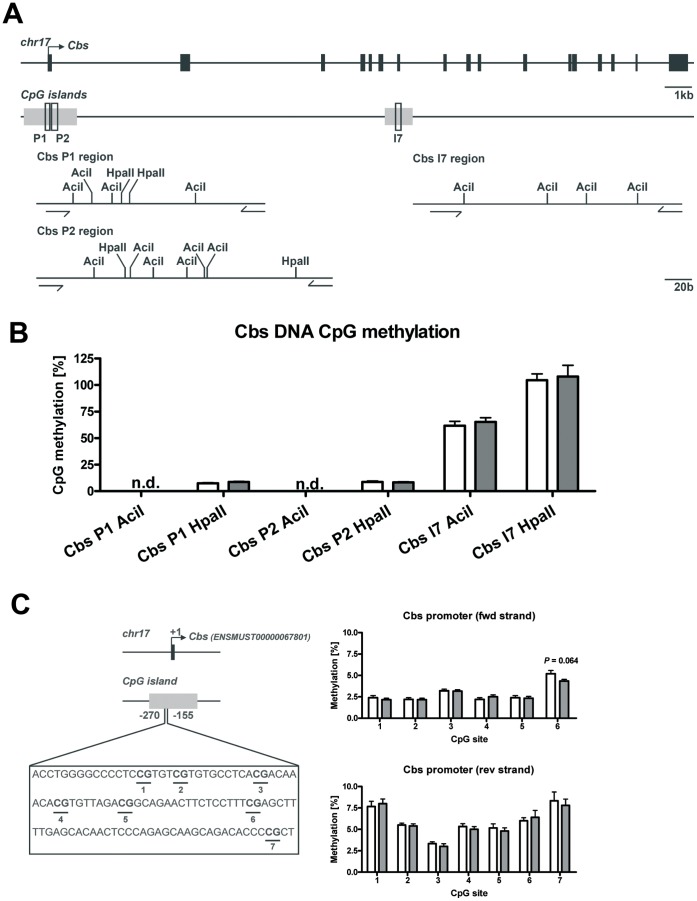
DNA methylation analysis in Cbs gene promoter and intragenic CpG islands of hepatic genomic DNA. Data are presented as mean ± SEM. Open and grey bars represent control and HF animals, respectively. Asterisk indicates statistical significance (p<0.05). From top to bottom are shown: (**A**) Cbs gene organization, localization of analyzed Cbs CpG islands in regions P1, P2 and I7 and corresponding Cbs CpG island restriction maps of informative restriction sites for MS-qPCR using the methylation sensitive restriction enzymes *Aci*I and *Hpa*II. (**B**) Analysis of DNA methylation of methylation-sensitive restriction sites in Cbs CpG islands of control and obese mice (n = 6). n.d., not detectable. (**C**) Analysis of DNA methylation of Cbs promoter CpG-island DNA methylation assessed by bisulfite genomic pyrosequencing. **Left:** Experimental design is depicted. Filled black box represents exon 1 and black arrow indicates transcription start site (ENSMUST00000067801). Filled grey box represent Cbs promoter CpG-islands and open box present the investigated nucleotide sequence (−270 until −155). The putative insulin response element (*PEPCK-like [TGTTTGT] motif*) on the reverse strand is flanked by the CpG #3 and CpG #4 on the forward strand (*PEPCK-like motif sequence on forward strand [ACAAACA]*). **Right:** Quantification of Cbs promoter CpG methylation (forward and reverse strand).

To investigate whether DIO influences epigenetic processes in the liver, DNA methylation, gene expression of DNA methyltransferases (Dnmt) as well genomic DNA methylation were determined. Animals receiving the C diet showed stronger expression for Dnmt1 and *de novo* Dnmt3a than for *de novo* Dnmt3b. Upon HF diet feeding, Dnmt1 mRNA expression was unaltered, but gene expression of Dnmt3a and Dnmt3b decreased by 25% and 54%, respectively (**Fig. 6**). However, global genomic DNA methylation was not affected (p = 0.87) as shown in **Figure 6D**. Previously, the promoter region of the rat Cbs gene was identified to be sensitive for DNA methylation [34] suggesting that the observed lower Cbs gene expression in our study could be accompanied by local genomic DNA methylation. Therefore, we measured the CpG methylation state in the CpG island of the Cbs promoter in the region P1 and P2 and of the intragenic CBS CpG island in the region I7 (**Fig. 7A**) with MS-qPCR. Analysis of hepatic DNA methylation in the CpG island regions P1 and P2 of the Cbs promoter in control and HF mice revealed no detectable or low CpG methylation of *Aci*I and *Hpa*II restriction sites (Cbs promoter *Hpa*II methylation; 8.08±0.54% in control animals and 8.46±0.37% in animals on HF diet) whereas the *Aci*I restriction site analysis of the Cbs intragenic region I7 showed CpG methylation of 61.68±4.25% in control liver genomic DNA and 65.24±3.95% in genomic DNA of HF diet liver samples. However, no significant differences of the investigated CpG sites between the groups (Cbs P1 *Hpa*II, p = 0.19; Cbs P2 *Hpa*II, p = 0.72; Cbs I7 *Aci*I, p = 0.55) could be observed as shown in **Figure 7B**. In addition, we analyzed 115 bp of the Cbs promoter CpG island (−270 until −155) containing a putative insulin response element (*PEPCK-like [TGTTTGT] motif*) [35] on the reverse strand flanked by CpGs by bisulfite conversion and subsequent pyrosequencing. The specific methylation analysis revealed only low CpG-methylation with no differences between the experimental groups except for a marginal decrease in methylation of CpG-site #6 on the forward strand (p = 0.064) as shown in **Figure. 7C**.

## Discussion

DIO in mice leads to NAFLD that is characterized by hepatic accumulation of fat which could result from increased fatty acid uptake, increased hepatic *de novo* fatty acid synthesis, impaired oxidation of fatty acids or a reduced VLDL-mediated TG export [36]. The latter is dependent on provision of sufficient PC for assembly of VLDL particles. It has been estimated that up to 30% of the hepatic PC biosynthesis originates from the PEMT pathway by a PEMT-mediated methylation of PE using SAM as a methyl-donor [9], [37], [38]. Previously, we have shown that feeding C57BL/6N mice a beef tallow-based HF diet resulted in hyperglycemia, hyperinsulinemia, reduced glucose tolerance and hepatic TG accumulation [5]. Furthermore, we have identified changes in the hepatic PL content and PC signature suggesting a DIO-based modulation of PC [5]. Recently, Rubio-Aliaga *et al.* also described alterations of hepatic C1-metabolism in mice upon HF diet feeding [21] demonstrating a general link between DIO- induced NAFLD and the C1-metabolism.

**Figure 8:**
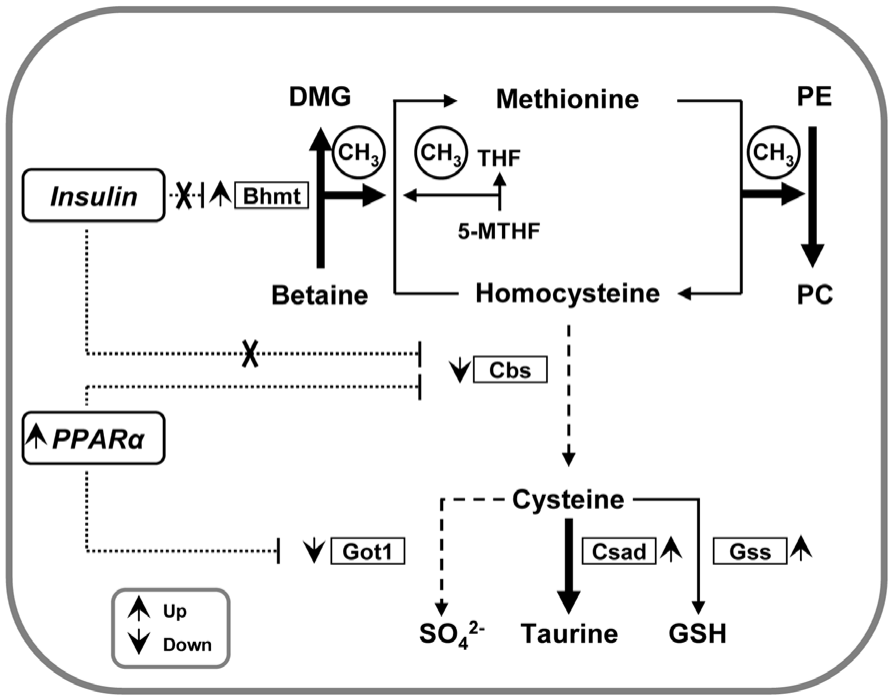
Schematic presentation of analyzed changes in hepatic C1-metabolism after HF feeding in C57BL/6N mice. Observed changes of mRNAs (Bhmt, Cbs, Csad, Got, Gss, PPARα), proteins (BHMT and CBS) and measured metabolites (taurine, homocysteine, methionine, betaine, DMG) are depicted. Dotted lines represent inhibitory effects of insulin (reported by [48], [49]) and PPARα (this study) on the regulation of transcription. Cross means disrupted inhibitory effect of insulin reported in hyperglycemic mice (40–43).

We here extend these observations and provide evidence that hepatic methionine homeostasis is conserved in C57BL/6N mice on a HF diet based on a PPARα-mediated downregulation of the hepatic transsulfuration pathway and an enhanced Hcy remethylation capacity associated with an elevated taurine production despite repression of hepatic transsulfuration (**Fig. 8**). These findings are compatible with data of Finkelstein *et al*. using rats fed a low methionine diet (0.25% w/w) with provision of extra dietary cystine (0.8% or 1.3% w/w) [39], [40]. In these rats, a hepatic methionine-sparing effect with reduced CBS activity accompanied with increased BHMT activity was observed. The lower CBS activity could only be detected under a low dietary methionine supply [39]. Our findings in mice fed a HF diet with sufficient methionine (0.86%, w/w) and cystine (0.46%, w/w) suggest that, despite an adequate methionine supply, the transsulfuration pathway is downregulated whereas BHMT-dependent Hcy-remethylation is increased. Furthermore, we detected lower mRNA levels of CBS and an increase of BHMT mRNA with corresponding changes on protein level. Tang *et al.* also identified a comparable mechanism in methionine-deprived C57BL/6 mice with a proposed post-transcriptional downregulation of hepatic CBS and increased BHMT enzyme activity associated with a transient increase in plasma Hcy level [41]. Interestingly, we detected a reduced Hcy transsulfuration and an increased Hcy remethylation capacity. This finding is supported by reduced hepatic betaine concentration, increased BHMT protein expression and increased [DMG]/[betaine] ratio in HF mice, suggesting increased BHMT activity. In rats, Finkelstein *et al*. showed that hepatic betaine content is influenced by Hcy remethylation and is dependent on dietary protein level [42], whereas the protein intake of control and HF mice in our study was not significantly different (**Table S2**). Our finding of reduced hepatic betaine concentrations in obese mice is in line with data of Kim *et al*. reporting significantly lower betaine levels in livers from DIO mice [43]. In general, the increased hepatic BHMT protein expression, [DMG]/[betaine] ratio and reduced betaine content in our HF mice are indicative for an enhanced BHMT mediated Hcy-remethylation, however we did not find any evidence for changes in the hepatic methionine cycle in obese mice. This implies that maintaining methionine levels by reduced Hcy transsulfuration and enhanced Hcy remethylation is of prime importance for hepatocytes in NAFLD despite elevated taurine levels derived from either hepatic or extrahepatic cysteine. Since methionine is an essential amino acid, a reduction in methionine levels would critically affect protein synthesis capacity for the maintenance of the hepatic proteome as well as its protein secretion function.

The development of liver steatosis upon HF feeding is known to be associated with hepatic insulin resistance [44], [45], [46] and enhanced rates of gluconeogenesis. HF mice analyzed in the present study exhibited similar characteristic phenotypic alterations as described previously [5]. In Zucker diabetic fatty rats representing a type 2 diabetes model and in a type 1 diabetes animal model with streptozotocin an impaired insulin secretion and signaling and an increased hepatic gene expression of CBS and BHMT representing the branch-point enzymes of C1-metabolism have been reported [47], [48], [49], [50]. This is to some extent in line with our data suggesting increased Hcy remethylation and improved VLDL secretion due to increased BHMT expression in obese animals [51]. Furthermore, the implication of BHMT in lipid and PL metabolism of the liver and adipose tissue was recently shown by Teng *et al.* [52], [53]. However, the identified downregulation of CBS in our study contradicts a disturbed insulin signaling as described by others [47], [48], [50] indicating that other mechanisms may have caused the observed decrease of CBS expression. In this context, reduced hepatic Cbs mRNA level and a reduced enzyme activity were also reported for rats fed a HF diet [54], [55] or for rats on HF diets treated with the PPARα agonist WY14,643 [56]. Our data also provide evidence for alterations in PPARα signaling in the liver of obese mice as shown by elevated mRNA expression of Pparα in conjunction with enhanced expression of prototypical PPARα target genes such as Cpt1a, Ucp2 or Acox1 (**Fig. 5A, B**). Although PPARα is primarily considered to affect genes controlling lipid metabolism, it is also a regulator of hepatic amino acid metabolism [33]. For example, Kersten *et al.* have shown that PPARα can mediate suppressive effect on urea cycle enzymes [57], and inhibitory effects of fatty acids, which are ligands of PPARα, on ureagenesis [58] and ammonia detoxification [59] have been described. In this context, interestingly, we detected higher concentrations for glutamine and lower citrulline and ornithine concentrations in the liver of HF mice than in controls pointing to changes in ureagenesis and ammonia detoxification upon prolonged HF feeding. In addition, glutamine is known to influence fatty acid oxidation, lipolysis and glutathione biosynthesis [60], [61], [62]. Regarding to the mechanistic role of PPARα in our study, we could demonstrate in hepatoma cells using the PPARα agonist WY14,643 that Cbs is a target gene of the PPARα signaling pathway (**Fig. 5B**) leading to a downregulation of the transsulfuration pathway comparably as shown by low CBS levels in HF mice. Interestingly, *in vitro* studies in HepG2 cells show decreased PPARα expression dependent on Hcy concentration, establishing a possible feedback signaling of the methionine cycle on PPARα signaling via Hcy [17], [63]. Findings from CBS-deficient mice [64], [65], [66] also corroborate the prominent role of the transsulfuration pathway and especially of CBS in the development of a fatty liver.

Taken together, mice fed a HF diet display an altered equilibrium between the transmethylation (methionine cycle) and processes that relate to the transsulfuration pathway such as glutathione and taurine synthesis which seem at least partially controlled by PPARα. The decrease in hepatic L-α-amino-n-butyrate levels found in our DIO mice suggest that α-ketobutyrate production from cystathionine in the transsulfuration pathway is also reduced which could affect also hepatic ophthalmic acid levels. This alternative non-thiol is produced by glutathione-synthetase when using L-α-amino-n-butyrate rather than cysteine as a substrate [67]. Reduced levels of L-α-amino-n-butyrate would promote increased glutathione levels for proper redox-balance in liver. The increased taurine levels observed in DIO mice may also be indicative for changes in stress-response. The observed downregulation of Got1 gene expression known to be regulated by PPARα activity [57] together with increased gene expression of Csad and elevated hepatic taurine concentrations in obese mice points to a reduced sulfate production from cysteine compensating reduced Hcy transsulfuration in favor of increased taurine synthesis. These changes may possibly improve osmoregulatory, cytoprotective and antioxidant capacities in the steatotic liver [68],[69]. In the nucleus, the principal methyl-donor, SAM, provides methyl-groups for DNA methylation and histone modification which are important for epigenetic gene expression regulation, chromatin condensation and genome integrity [70], [71], [72]. Although we observed a downregulation of hepatic *de novo* Dnmts, this did not seem to influence the global DNA methylation state in our DIO mice. Although, in male Sprague-Dawley rats, diabetes-mediated perturbations in C1-metabolism resulted in hepatic DNA-hypomethylation we could not confirm this in our DIO mice [73]. The analysis of CpG island DNA methylation of the Cbs gene by MS-qPCR and analyzing 115 bp of a Cbs promoter CpG island (−270 until −155) containing a putative insulin response element (*PEPCK-like [TGTTTGT] motif*) [35] by bisulfite conversion/pyrosequencing of liver genomic DNA from DIO mice compared with controls revealed no obvious changes in DNA methylation in the Cbs promoter or intragenic region. However, our DNA methylation analysis along the Cbs gene by MS-qPCR may have not detected DNA methylation due to the limited detection sensitivity of the MS-qPCR method at CpG sites in specific DNA sequence areas or in case of bisulfite conversion/pyrosequencing anaylsis with lower methylation frequency compared to bisulfite-sequencing shown by Uekawa *et al.* [34].

In conclusion, our data demonstrate that HF diet feeding in mice can induce a suppression of gene and protein expression of enzymes operating in the hepatic transsulfuration pathway in favor of an increased remethylation of Hcy to methionine. In addition, we show that the PPARα pathway is involved in the downregulation of CBS. Based on our findings, we postulate the primacy of methionine homeostasis mediated by remethylation of Hcy to methionine in liver physiology and in pathological situations such as NAFLD to ensure the maintenance of basic liver functions such as synthesis of hepatic and plasma proteins despite previously reported altered PL homeostasis and PC signature. High dietary fat intake associated with the development of hepatic steatosis is consequently linked to major changes in hepatic C1-metabolism that secondarily may also translate into changes of hepatic redox-status and cellular osmolyte levels.

## Supporting Information

Table S1 **Composition of experimental diets.**
(PDF)

Table S2 **Effect of HF diet feeding on nutrient and energy intake of experimental mice.**
(PDF)

Table S3 **Mouse primer sequences for gene expression analysis in tissue.**
(PDF)

Table S4 **Primer sequences for gene expression analysis in rat hepatoma cells (Fao).**
(PDF)

Table S5 **MS-qPCR primer sequences for local DNA methylation analysis.**
(PDF)

Table S6 **List of primer sequences used for quantification of individual CpG-site DNA methylation analysis by bisulfite genomic pyrosequencing.**
(PDF)

Table S7 **Effect of HF feeding on hepatic metabolite concentrations.**
(PDF)
