# Correction: Hepatic Methionine Homeostasis Is Conserved in C57BL/6N Mice on High-Fat Diet Despite Major Changes in Hepatic One-Carbon Metabolism

**DOI:** 10.1371/annotation/9dd8c2df-1921-45b4-b3ba-8919f9068aea

**Published:** 2013-10-16

**Authors:** Christoph Dahlhoff, Charles Desmarchelier, Manuela Sailer, Rainer W. Fürst, Alexander Haag, Susanne E. Ulbrich, Björn Hummel, Rima Obeid, Jürgen Geisel, Bernhard L. Bader, Hannelore Daniel

There were errors in the author affiliations. The correct affiliations should appear as shown here:

Christoph Dahlhoff 1,2

Charles Desmarchelier 1

Manuela Sailer 1

Rainer W. Fürst 2,4

Alexander Haag 1

Susanne E. Ulbrich 2,4

Björn Hummel 5

Rima Obeid 5

Jürgen Geisel 5

Bernhard L. Bader 2,3

Hannelore Daniel 1

1 Biochemistry Unit, ZIEL-Research Center for Nutrition and Food Sciences, Technische Universität München, Freising-Weihenstephan, Germany

2 PhD Group-Epigenetics, Imprinting and Nutrition, ZIEL-Research Center for Nutrition and Food Sciences, Technische Universität München, Freising Weihenstephan, Germany

3 Nutritional Medicine Unit, ZIEL-Research Center for Nutrition and Food Sciences, Technische Universität München, Freising-Weihenstephan, Germany

4 Physiology Unit, ZIEL-Research Center for Nutrition and Food Sciences, Technische Universität München, Freising-Weihenstephan, Germany

5 Clinical Chemistry and Laboratory Medicine/Central Laboratory, University Hospital of the Saarland, Homburg, Germany 

