# Supplementary material for: Hepatic Methionine Homeostasis Is Conserved in C57BL/6N Mice on High-Fat Diet Despite Major Changes in Hepatic One-Carbon Metabolism
Source: PLoS One. 2013 Mar 5;8(3):e57387. doi: 10.1371/journal.pone.0057387 (PMC3589430; doi:10.1371/journal.pone.0057387)
Supplement: Table S1 — Composition of experimental diets. (PDF) [file pone.0057387.s001.pdf]

Table S1. Composition of experimental diets.

	C	HF
**Dry matter**	952	971
**Crude protein**	208	241
**Crude fat**	42	340
**Butter fat**	-	-
**Beef tallow**	-	310
**Soybean oil**	40	30
**Coconut fat**	-	-
**Cocoa butter**	-	-
**Crude fiber**	50	60
**Crude ash**	56	61
**Starch**	468	22
**Sugar/dextrine**	108	224
**N-free extracts**	594	270
**GE [MJ/kg]**	18	25.2
**ME [MJ/kg]**	15	21.4
**% carbohydrate**	66	21
**% protein**	23	19
**% fat**	11	60
Amino acids		
**Lysine**	17.1	19.8
**Methionine**	7.3	8.3
**Cystine**	0.9	4.6
**Methionine + Cystine**	8.2	12.8
**Threonine**	9.3	10.7
**Tryptophan**	2.7	3.1
**Arginine**	7.6	8.8
**Histidine**	6.6	7.6
**Valine**	14.2	16.4
**Isoleucine**	10.9	12.5
**Leucine**	20.5	23.6
**Phenylalanine**	11.1	12.9
**Phenylalanine + Tyrosine**	22.2	25.7
**Glycine**	4.3	5.0
**Glutamic acid**	46.9	54.1
**Aspartic acid**	15.5	17.9

Table S1 continued

Amino acids	C	HF
**Proline**	23.9	27.6
**Alanine**	6.8	7.9
**Serine**	12.4	14.3
Vitamins		
**Vitamin A**	15000 IU	15000 IU
**Vitamin D3**	1500 IU	1500 IU
**Vitamin E**	150 mg/kg	150 mg/kg
**Vitamin K (as menadione)**	20mg/kg	20mg/kg
**Vitamin C**	30 mg/kg	30 mg/kg
**Thiamin (B1)**	16 mg/kg	16 mg/kg
**Riboflavin (B2)**	16 mg/kg	16 mg/kg
**Pyridoxine (B6)**	18 mg/kg	18 mg/kg
**Cobalamin (B12)**	30 µg/kg	30 µg/kg
**Nicotinic acid**	49 mg/kg	45 mg/kg
**Pantothenic acid**	56 mg/kg	55 mg/kg
**Folic acid**	19 mg/kg	19 mg/kg
**Biotin**	310 µg/kg	310 µg/kg
**Choline-Chloride**	1.04 g/kg	2.3 g/kg
**Inositol**	80 mg/kg	80 mg/kg
**Betaine**	-	-
Trace elements		
**Iron**	166 mg/kg	139 mg/kg
**Manganese**	98 mg/kg	82 mg/kg
**Zinc**	65 mg/kg	56 mg/kg
**Zinc sulfate**	-	-
**Copper**	14 mg/kg	12 mg/kg
**Iodine**	1.2 mg/kg	0.97 mg/kg
**Selenium**	0.14 mg/kg	0.13 mg/kg
**Cobalt**	0.15 mg/kg	0.13 mg/kg
Fatty acids [% in diet]		
**C12:0**	-	0.03
**C14:0**	0.02	1.03
**C16:0**	0.45	8.06
**C17:0**	-	0.38
**C18:0**	0.19	5.61
**C20:0**	0.02	0.04
**C16:1**	0.02	0.78
**C18:1**	1.07	12.13
**C20:1**	-	0.01

Table S1 continued

Fatty acids [% in diet]	C	HF
**C18:2**	2.12	2.37
**C18:3**	0.26	0.33
**C20:4**	-	0.07

Nutrient compositions are reported by the manufacturer and are expressed as [g / kg], except it is delineated differently. The composition of the standard laboratory chow used for two weeks prior to the dietary intervention was: dry matter, 877; crude protein, 190; crude fat, 33; crude fiber, 49; crude ash, 64; starch, 365; sugar/dextrines, 47; N-free extracts, 541; GE, 16.3; ME, 12.8; % carbohydrate, 58; % protein, 33; % fat, 9. Abbreviations: GE, gross energy; ME, metabolizable energy calculated with the Atwater factors; N, nitrogen.
