# Supplementary material for: Hepatic Methionine Homeostasis Is Conserved in C57BL/6N Mice on High-Fat Diet Despite Major Changes in Hepatic One-Carbon Metabolism
Source: PLoS One. 2013 Mar 5;8(3):e57387. doi: 10.1371/journal.pone.0057387 (PMC3589430; doi:10.1371/journal.pone.0057387)
Supplement: Table S2 — Effect of HF diet feeding on nutrient and energy intake of experimental mice. (PDF) [file pone.0057387.s002.pdf]

Table S2. Effect of HF diet feeding on nutrient and energy intake of experimental mice.

	**C**	**HF**
**Food intake [g]**	306.6 ± 2.4	262.5 ± 2.3*
**Energy intake [kcal]**	1317.9 ± 10.3	1579.9 ± 13.7*
**Protein intake [g]**	63.8 ± 0.5	63.3 ± 0.55
**Methionine intake [g]**	2.33 ± 0.02	2.26 ± 0.02*
**Cystine intake [g]**	0.28 ± 0.002	1.21 ± 0.01*
**Choline chloride intake [g]**	0.319 ± 0.003	0.604 ± 0.005*
**Folate intake [mg]**	6.13 ± 0.05	5.25 ±0.05*
**Vitamin B_{12} intake [µg]**	9.20 ± 0.07	7.88 ± 0.07*

Data are presented as mean ± SEM (n = 9 - 11). Asterisk indicates statistical significance ($p < 0.05$).
