# Supplementary material for: Hepatic Methionine Homeostasis Is Conserved in C57BL/6N Mice on High-Fat Diet Despite Major Changes in Hepatic One-Carbon Metabolism
Source: PLoS One. 2013 Mar 5;8(3):e57387. doi: 10.1371/journal.pone.0057387 (PMC3589430; doi:10.1371/journal.pone.0057387)
Supplement: Table S3 — Mouse primer sequences for gene expression analysis in tissue. (PDF) [file pone.0057387.s003.pdf]

Table S3. Mouse primer sequences for gene expression analysis in tissue.

Symbol	Gene	Forward primer (5' to 3')	Reverse primer (5' to 3')
**Acox1**	Acyl-coenzyme A oxidase 1	CCAATCATGCGATAGTCCTGGC	CTTCAGGTAGCCATTATCCATCTC
**Actb**	Actin, beta	CCACTGCCGCATCCTCTTCC	GCCACAGGATTCCATACCCAAGA
**Ahcy**	S-adenosylhomocysteine hydrolase	CAGCTTCTGTCAGGCATCCG	AACTTGCTCTTGGTGACAGAATCG
**Bhmt**	Betaine-homocysteine methyltransferase	GGAGGCTGCGCGGTTGAAA	CTAGTGGCAACTCGGGGTTCC
**Bhmt2**	Betaine-homocysteine methyltransferase 2	CAT CCA AGT GCA GTT CGT CAA CT	TGC ATT CAC AGC TTC CCA CTT G
**Cbs**	Cystathionine β-synthase	CACAGTGCTGACCAAATCCCCC	CACACTTGGCCAAGAGCTCAC
**Chdh**	Choline dehydrogenase	CACGTCAGGCTGGCTACCC	GCAAGTAGGCGCAGGCTG
**Cpt1a**	Carnitine palmitoyltransferase 1a	GTCCCAGCTGTCAAAGATACCG	ATGGCGTAGTAGTTGCTGTTAACC
**Csad**	Cysteine sulfinic acid decarboxylase	GAGGACCTGGAGAGGCAGATC	CAAACATCGGCAATTGCATCCAG
**Cth**	Cystathionase	GCTAGAGGCAGCGATTACACC	GCAGACATGAAGGTGTTATCTACAACC
**Dmgdh**	Dimethylglycine dehydrogenase precursor	GGCACGCAGCAGGTTTAACAAC	GGAATCCCACCACCTGTCCGG
**Dnmt1**	DNA methyltransferase 1	GGAAGGCTACCTGGCTAAAGTCAAG	GGGTGTCACTGTCCGACTTGC
**Dnmt3a**	DNA methyltransferase 3A	TGGAGAATGGCTGCTGTGTGAC	CACTCATCCCGTTTCCGTTTG
**Dnmt3b**	DNA methyltransferase 3B	AGTGACCAGTCCTCAGACACGAAG	ATCAGAGCCATTCCCATCATCTAC
**Gapdh**	Glyceraldehyde-3-phosphate dehydrogenase	CCTGGAGAAACCTGCCAAGTATG	GAGTGGGAGTTGCTGTTGAAGTC
**Gclc**	Glutamate-cysteine ligase, catalytic subunit	AGCATCAGGCTCTCTGCACC	TTCTCCTCTCCGATGCCGG
**Gnmt**	Glycine N-methyltransferase	GCCAGACTGCAAAGGTGACCA	GTCGTAATGTCCTTGGTCAGGTCA
**Got1**	Glutamate oxaloacetate transaminase 1, soluble	GAGCTGTGCTTCTCGCCTAG	TCCCAGGTTGGTGATGATACGTAG
**Gss**	Glutathione synthetase	CCTATGCTGTGCAGATGGACTTC	CAAAGAGACGGGCAGTATAGTCG
**Hprt**	Hypoxanthine guanine phosphoribosyl transferase	GTCGTGATTAGCGATGATGAACC	GTCTTTCAGTCCTGTCCATAATCAG

Table S3 continued

Symbol	Gene	Forward primer (5’ to 3’)	Reverse primer (5’ to 3’)
**Mat1a**	Methionine adenosyltransferase I, alpha	GTGGCCTGTGAGACAGTGTGC	TGTCTCTCACCACCCGCTGG
**Mat2a**	Methionine adenosyltransferase II, alpha	AAGTGGCTTGTGAAACTGTTGCT	CTTGGGCAATATCTGGTGACTGTTG
**Mthfr**	5,10-methylenetetrahydrofolate reductase	GACATCTGTGTGGCAGGTTACCC	CTGAAGAAGGTGCTGGCCTC
**Mtr**	5-methyltetrahydrofolate-homocysteine methyltransferase	GCTGGTGGACTACATTGACTGGA	TTCTGGCTTCTTCACCTACTGC
**Pemt**	Phosphatidylethanolamine N-methyltransferase	CTGGAATGTGGTAGCGAGATGG	CAGTGGGAGCGGAGGATGTTC
**Ppara**	Peroxisome proliferator activated receptor α	CCAGTACTTAGGAAGCTGTCCG	TATTCGACACTCGATGTTCAGGG
**Shmt1**	Serine hydroxymethyltransferase 1 (soluble)	CTGCGGAAGATTGCGGATGAT	GTCTTGGGATCCACACTGCGC
**Shmt2**	Serine hydroxymethyltransferase 2 (mitochondrial)	CCTATGCCCGCCTCATTGACT	TTCCGGTAGAAGATGAGCCCTG
**Ucp2**	Uncoupling protein 2	GCAGATCCAAGGGGAGAGTCAA	CCGGCGACCAGCCCATTG
