# Supplementary material for: Hepatic Methionine Homeostasis Is Conserved in C57BL/6N Mice on High-Fat Diet Despite Major Changes in Hepatic One-Carbon Metabolism
Source: PLoS One. 2013 Mar 5;8(3):e57387. doi: 10.1371/journal.pone.0057387 (PMC3589430; doi:10.1371/journal.pone.0057387)
Supplement: Table S4 — Primer sequences for gene expression analysis in rat hepatoma cells (Fao). (PDF) [file pone.0057387.s004.pdf]

Table S4. Primer sequences for gene expression analysis in rat hepatoma cells (Fao).

Symbol	Gene	Forward primer (5' to 3')	Reverse primer (5' to 3')
**Bhmt**	Betaine-homocysteine methyltransferase	AGGCTGCGGTGGAGCACC	AGATATCTTCTCTGCCACGTAGTTC
**Cbs**	Cystathionine β-synthase	CGTAGGAGTGGCATGGCGAC	TTCCCGTCGCACTGCTGC
**Cpt1a**	Carnitine palmitoyltransferase 1a	GAGCCGCAGCACCAAGATC	TCTTCCTTCATCAGTGGCCTCAC
**Gus**	β-glucuronidase	GCATGAGCATCCAGCCTACC	GAACTTGCTCTTTGTGACAGCC
