# Supplementary material for: Hepatic Methionine Homeostasis Is Conserved in C57BL/6N Mice on High-Fat Diet Despite Major Changes in Hepatic One-Carbon Metabolism
Source: PLoS One. 2013 Mar 5;8(3):e57387. doi: 10.1371/journal.pone.0057387 (PMC3589430; doi:10.1371/journal.pone.0057387)
Supplement: Table S5 — MS-qPCR primer sequences for local DNA methylation analysis. (PDF) [file pone.0057387.s005.pdf]

Table S5. MS-qPCR primer sequences for local DNA methylation analysis.

Symbol	Primer name	Forward primer (5’ to 3’)	Reverse primer (5’ to 3’)
**Chr18***	Chromosome 18	GCAATCAGGCTTGTAGCAGTT	CATATGCACCATGTGTCTTGG
**Cbs P1**	Cystathionine β-synthase promoter region 1	GGTGAGTGGGAGGCCGTTCT	TGGAGAGCACGCTCGGAC
**Cbs P2**	Cystathionine β-synthase promoter region 2	GTCCGAGCGTGCTCTCCA	TTAGCGAGCTGCGCGTC
**Cbs I7**	Cystathionine β-synthase intragenic region 7)	GCTGCTTACTGTAGGACTTGTTG	GCACCGTCACACTGCTGC
**Zrsr1***	Zinc finger (CCCH type), RNA binding motif and serine / arginine rich 1	GGCCGTACCACAGATAACCA	GCGCAGTTATCCGTCTATCCA

* Primer sequences as used by Oakes et al. [28].
