# Supplementary material for: Hepatic Methionine Homeostasis Is Conserved in C57BL/6N Mice on High-Fat Diet Despite Major Changes in Hepatic One-Carbon Metabolism
Source: PLoS One. 2013 Mar 5;8(3):e57387. doi: 10.1371/journal.pone.0057387 (PMC3589430; doi:10.1371/journal.pone.0057387)
Supplement: Table S6 — List of primer sequences used for quantification of individual CpG-site DNA methylation analysis by bisulfite genomic pyrosequencing. (PDF) [file pone.0057387.s006.pdf]

Table S6. Primer sequences used for quantification of individual CpG-site DNA methylation analysis by bisulfite genomic pyrosequencing.

Symbol	Primer name	Forward primer (5'-3')	Reverse primer (5'-3')
**Cbs fst**	Cbs forward strand	AGGTTGTGTAGTAAGGTAT AGATTTTTGGGTATAG	**B-** CTCTCCACCCCCACTCTA ACTC
**Cbs fst seq**	Cbs forward strand sequencing primer	GGTATAGATTTGGGGTTTT	
**Cbs rst**	Cbs reverse strand	TTTTATAGAGGGGTGTTTG TTTGTTTTGGGAGTTG	**B-** ACTAACCCAAACCTCTCA AACCAATTACTT
**Cbs rst seq**	Cbs reverse strand sequencing primer	TTGTTTTGGGAGTTGT	

fst, forward genomic DNA strand; rst, reverse genomic DNA strand; B, biotinylation.
