# Supplementary material for: Hepatic Methionine Homeostasis Is Conserved in C57BL/6N Mice on High-Fat Diet Despite Major Changes in Hepatic One-Carbon Metabolism
Source: PLoS One. 2013 Mar 5;8(3):e57387. doi: 10.1371/journal.pone.0057387 (PMC3589430; doi:10.1371/journal.pone.0057387)
Supplement: Table S7 — Effect of HF feeding on hepatic metabolite concentrations. (PDF) [file pone.0057387.s007.pdf]

Table S7. Effect of HF feeding on hepatic metabolite concentrations.

Metabolite [µmol/g protein]	C	HF
**1MHis**	0.36 ± 0.02	0.47 ± 0.05
**Aad**	0.52 ± 0.07	0.56 ± 0.08
**Abu**	0.96 ± 0.14	0.47 ± 0.04*
**Ala**	70.30 ± 3.48	77.87 ± 4.21
**Asn**	2.39 ± 0.16	2.72 ± 0.18
**Asp**	3.20 ± 0.27	2.71 ± 0.30
**bAib**	0.13 ± 0.01	0.10 ± 0.01
**Cit**	0.71 ± 0.08	0.38 ± 0.03*
**Cys**[a]	0.28 ± 0.02	0.27 ± 0.03
**EtN**	0.35 ± 0.02	0.33 ± 0.03
**GABA**	0.22 ± 0.01	0.23 ± 0.02
**Gln**	38.43 ± 2.95	53.07 ± 3.71*
**Glu**	13.96 ± 0.77	18.19 ± 1.91
**Gly**	32.00 ± 1.16	28.75 ± 1.23
**His**	7.99 ± 0.57	7.91 ± 0.61
**Hyp**	0.34 ± 0.02	0.24 ± 0.02*
**Ile**	4.13 ± 0.32	3.80 ± 0.29
**Leu**	7.03 ± 0.55	6.53 ± 0.48
**Lys**	9.04 ± 0.71	7.69 ± 0.51
**Met**	1.37 ± 0.26	0.96 ± 0.24
**Orn**	3.65 ± 0.31	2.64 ± 0.19*
**PEtN**	3.60 ± 0.23	3.98 ± 0.36
**Phe**	2.18 ± 0.19	2.41 ± 0.21
**Pro**	5.69 ± 0.80	5.57 ± 0.52
**Sar**	0.88 ± 0.15	0.83 ± 0.03
**Ser**	3.48 ± 0.33	3.57 ± 0.27
**Tau**	125.56 ± 4.31	158.38 ± 6.68*
**Thr**	4.43 ± 0.49	4.40 ± 0.34
**Trp**	0.66 ± 0.05	0.65 ± 0.05
**Tyr**	2.51 ± 0.13	2.22 ± 0.19
**Val**	7.84 ± 0.56	6.95 ± 0.54

Data are presented as mean ± SEM (n = 9 - 11). Asterisk indicates statistical significance ($p < 0.05$).
a, below quantification limit. Abbreviations: 1MHis, 1-methyl-L-histidine; Aad, L-α-aminoadipic acid; Abu, L-α-amino-n-butyrate; Ala, L-alanine; Asn, L-asparagine; Asp, L-asparic acid; bAib, D,L-β-aminoisobutyrate; Cit, L-citrulline; Cys, L-cysteine; EtN, ethanolamine; GABA, γ-amino-n-butyrate; Gln, L-glutamine; Glu, L-glutamate; Gly, glycine; His, L-histidine; Hyp, hydroxyproline; Ile, L-isoleucine; Leu, L-leucine; Lys, L-lysine; Met, L-methionine; Orn, L-ornithine; PEtN, O-phosphoethanolamine; Phe, L-phenylalanine; Pro, L-proline; Sar, sarcosine; Ser, L-serine; Tau, taurine; Thr, L-threonine; Trp, L-tryptophane; Tyr, L-tyrosine; Val, L-valine.
